# Eviction, post-traumatic stress, and emergency department use among low-income individuals in New Haven, CT

**DOI:** 10.1016/j.pmedr.2022.101956

**Published:** 2022-08-17

**Authors:** Patrick D. Smith, Allison K. Groves, Brent A. Langellier, Danya E. Keene, Alana Rosenberg, Kim M. Blankenship

**Affiliations:** a Drexel University Dornsife School of Public Health, Department of Community Health and Prevention, Nesbitt Hall, 3215 Market Street, Philadelphia, PA 19104, USA; b Drexel University Dornsife School of Public Health, Department of Health Management and Policy, Nesbitt Hall, 3215 Market Street, Philadelphia, PA 19104, USA; c Yale University School of Public Health, Department of Social and Behavioral Sciences, 60 College Street, New Haven, CT 06510, USA; d American University, Department of Sociology, 4400 Massachusetts Avenue, Washington, DC 20016, USA

## Abstract

We sought to examine whether and how landlord-related forced moves (inclusive of, but not limited to, legal eviction) were associated with emergency department (ED) use over time.

We used survey data collected between 2017 and 2019 among 283 low-income participants in New Haven, CT to examine whether experiencing a legal eviction or other landlord-related forced move (T_0_) was associated with increased odds of ED use 6 months (T_1_) and 12 months (T_2_) later. We conducted bootstrapped mediation analyses to examine indirect effects of post-traumatic stress symptoms.

One-fifth of participants (n = 61) reported a recent forced move at baseline (T_0_); half of these were legally evicted. Landlord-related forced moves were associated with ED use at T_1 _(AOR = 2.06, 95 % CI: 1.04–4.06) and T_2_ (AOR = 3.05, 95 % CI: 1.59–5.88). After adjustment for sociodemographic factors and other health-related confounders, legal eviction was not significantly associated with ED use at T_1_ (AOR = 1.61, 95 % CI: 0.68–3.81), but was significantly associated with ED use at T_2_ (AOR = 3.58, 95 % CI: 1.58–8.10). Post-traumatic stress symptoms accounted for 15.1% of forced moves’ association with ED use (p <.05).

Landlord-related forced moves are positively associated with subsequent ED use, and post-traumatic stress symptoms are one factor that may help explain this association. Structural interventions that promote housing stability are needed to advance health equity, and they may also help to reduce preventable ED use. Such interventions are imperative in the context of the COVID-19 pandemic, which has strained health system capacity and exacerbated housing instability for many low-income renters. Results underscore the relevance of trauma-informed care and integrated care management to clinical practice in emergency settings.

## Highlights

• Past research indicates that eviction adversely impacts physical and mental health.
• Few studies have examined whether and how eviction impacts healthcare use.
• We found that legal and informal evictions were associated with subsequent ED use.
• We found that post-traumatic stress symptoms were common after eviction.
• Interventions to advance housing stability may improve health and decrease future ED use.

## 1 Introduction

Access to safe, affordable, and stable housing has been recognized as both a human right and a social determinant of health (Office of the High Commissioner for Human Rights, 1993, Wilkinson and Marmot, 2003). Despite this, more than two million American renters are legally evicted annually (Desmond et al., 2018). Furthermore, an even greater number of renters are forced to move through informal eviction processes – including eviction threats and abrupt rent increases – each year (Desmond and Shollenberger, 2015, Groves et al., 2021). Owing to the compounding effects of rising housing costs (Joint Center for Housing Studies, 2022), wage stagnation (Horowitz et al., 2020), insufficient rental assistance (Tars, 2018), and discrimination (Reskin, 2012, Rosen et al., 2021, Greenberg et al., 2016), structurally marginalized groups – including low-income and Black individuals – bear a disproportionately high burden of eviction filings and judgments (Desmond and Gershenson, 2017, Hepburn et al., 2020). While the rate of legal evictions decreased in response to federal and local eviction moratoria implemented during the COVID-19 pandemic, eviction filing rates are now returning to pre-pandemic levels in many localities (Eviction Lab, 2022). According to U.S. Census Bureau Pulse Surveys from June-July 2022, 14.7 % of renter households are behind on rent; and among households behind on rent, 49.7 % think an eviction is “very likely” or “somewhat likely” in the next two months (US Census Bureau, 2022).

While growing literature indicates that eviction adversely impacts physical and mental health across the life course (Vásquez-Vera et al., 2017) and that health-related effects of eviction may persist for years (Hatch and Yun, 2021), little is known about how legal eviction and other landlord-related forced moves (LRFM) impact healthcare use broadly, and emergency department (ED) use, specifically. Conceptually, eviction and other LRFMs may increase ED use for several reasons. Firstly, eviction increases risk of homelessness and housing instability (Desmond et al., 2015), which are independently associated with delayed usage of primary care services and increased use of emergency services (for medical needs and/or shelter) (Fazel et al., 2014, Khandor et al., 2011, Ma et al., 2008). Past research suggests that EDs are often a “first-stop” access site when persons initially become homeless (O’Toole et al., 2007), and a recent systematic review underscores that the association between homelessness and ED use persists beyond the inciting event (Salhi et al., 2018). Secondly, eviction frequently leads to the loss of one’s possessions (potentially including medications) and to financial strain (Desmond, 2016, Desmond and Gershenson, 2016, Humphries et al., 2019), which may exacerbate and/or disrupt management of chronic mental and physical health conditions (Osborn et al., 2017), precipitating a health emergency. Thirdly, ethnographic and empirical research indicates that eviction is a distinctly traumatizing incident that affects mental health (Desmond and Kimbro, 2015, Desmond, 2016, Hoke and Boen, 2021). In sum, eviction may lead to increased ED use, and the onset or exacerbation of mental health conditions may mediate this association. Effects of other landlord-related forced moves, such as those driven by eviction threats, may be similar; yet such moves are understudied in past research.

Underscoring the above, a recent study of adults in housing court in New York City found that eviction increased the number of ED visits by over 70 % in the two years after filing, while also increasing one’s likelihood of inpatient mental health hospitalization over the same period (Collinson and Reed, 2018). In another recent study of Medicaid-insured patients in New York, researchers found that use of acute care services (including ED visits) increased marginally in the six months following a court-ordered eviction (Schwartz et al., 2022). Outside this context, no other studies have examined how eviction and other forced moves are associated with ED use, including whether these associations vary over time. Further, no studies have explicitly examined mediating pathways through which eviction may influence healthcare use. Clarifying this relationship is critical to understanding the association between unmet housing needs and potentially preventable healthcare use.

Several additional gaps remain in research on eviction and health. Firstly, past studies have focused almost exclusively on health-related impacts of court-ordered eviction, despite evidence that court-ordered evictions account for fewer than one in four forced moves among U.S. renters (Desmond and Shollenberger, 2015, Gromis and Desmond, 2021). Conceptually, the unique stresses, stigma, and discrimination associated with the legal eviction process may produce particularly significant effects on post-traumatic stress and ED use, given that legal evictions create unique barriers to subsequent housing stability (Kleysteuber, 2007, Smith v. Wasatch Property Management, 2017). At the same time, all involuntary moves precipitated by a landlord may lead to significant stress and instability, thus underscoring the importance of examining whether associations between eviction and health persist when considering the broader construct of landlord-related forced moves. Secondly, while prior studies have identified associations between eviction and poor mental health (Desmond and Kimbro, 2015, Hatch and Yun, 2021, Hoke and Boen, 2021), few have used clinically-validated measures to examine whether eviction is associated with experiencing symptoms of specific mental health conditions, such as post-traumatic stress disorder (PTSD). Thirdly, several empiric studies on eviction’s health-related impacts have occurred in international settings (Damon et al., 2019, Kennedy et al., 2017, Pilarinos et al., 2017, Rojas, 2017, Rojas and Stenberg, 2016), where context-specific housing and health policies may limit generalizability to U.S. contexts.

Given gaps in research, a projected rise in forced moves following the withdrawal of COVID-19-related eviction moratoria, and concerns about rates of preventable healthcare use, further research is needed to discern whether and how eviction and other landlord-related forced moves are associated with ED use. Leveraging data from a longitudinal cohort study of adults in New Haven, CT, this study seeks to identify whether evictions are associated with future ED use, examine the durability of this association over time, and determine whether this association persists when considering landlord-related forced moves more broadly. We further explore whether experiencing post-traumatic stress symptoms partially explains the association between LRFMs and ED use. This study can strengthen prevention efforts and care delivery by highlighting the relationships between forced moves, mental health, and healthcare use.

## 2 Methods

### 2.1 Study design

Our analytic sample is drawn from the Justice, Housing, and Health Study (JustHouHS), a longitudinal cohort study designed to examine intersections between incarceration, housing, and sexual health among 400 adults in New Haven, CT. JustHouHS data were collected between 2017 and 2021; this analysis uses data collected between September 2017 and April 2019.

JustHouHS participants were recruited using flyers, outreach to local service providers, community meetings, and snowball sampling. Research staff screened interested participants (n = 616). Of those eligible (n = 471), 71 individuals did not enroll into JustHouHS. Participants were eligible if they (1) were ≥18 years; (2) were a resident of New Haven; (3) had no household members already in the study, and (4) were low-income, defined as meeting at least one of the following criteria: (a) received housing or food assistance in the past year, (b) were Medicaid recipients, (c) were homeless, or (d) resided in low-income census tracts, where >20 % of residents lived below the federal poverty line. Given the study’s focus on inter-relationships between housing, incarceration, and health, the total sample was stratified to include 200 individuals released from prison or jail in the past year and 200 who were not released in the past year. All participants provided written consent. Participants completed a self-administered computer-assisted survey at each study visit, for which they were compensated. Study activities were approved by the Yale University IRB.

At baseline, 400 participants enrolled in JustHouHS. Starting six months after baseline survey completion, participants completed four follow-up surveys, with high retention rates (>75 % for each wave). The current analyses included data from each participant’s baseline survey (T_0_) and first two follow-up surveys (T_1,_ T_2_). We used a complete case analysis approach, excluding participants who were missing data for exposures (T_0_), covariates (T_0_), mediator (T_1_), or outcomes (T_1_, T_2_). Specifically, we excluded 87 individuals missing outcome data at T_1_ and an additional 30 individuals missing outcome data at T_2_. We examined differences between our analytic sample and the broader JustHouHS sample using chi-square tests for categorical variables and t-tests for continuous variables. Compared to JustHouHS participants more broadly, those excluded from this analysis were more likely to be male, non-Latinx white, recently incarcerated, and non-high school graduates. Characteristics of included vs excluded individuals are documented in Supplemental Table 1.

## 3 Measures

### 3.1 Exposure

We separately examined two binary (yes/no) exposures: legal eviction and landlord-related forced moves.

Participants were coded as having a legal eviction (1) if they reported at baseline (T_0_) that they had been legally evicted any time in the past two years. All others were coded 0.

Participants were coded as having a landlord-related forced move (1) if they reported, at baseline: (a) a legal eviction and/or (b) that their most recent move, within the past 2 years, had occurred for any of the following reasons: eviction, rent increase, non-payment of rent, damage to rental unit, accusation of illegal activity, landlord stating too many people were living in the unit, or landlord foreclosure. All others were coded 0.

### 3.2 Outcome

The outcome variable was a binary (yes/no) measure of self-reported emergency department use within the past 6 months, measured separately at two time points: T_1_ (6 months post-baseline) and T_2_ (12 months post-baseline). Past research has established high degrees of concordance between self-report and administrative records of ED use within one year of service use (Short et al., 2009).

### 3.3 Mediator

Post-traumatic stress was measured using the *Primary Care Post-Traumatic Stress Disorder for DSM-5* scale *(PC-PTSD-5)*. The PC-PTSD-5 is a validated, five-item screener for symptoms of post-traumatic stress disorder (PTSD) in adults. We dichotomized this measure using a cut-point of 3, which is considered optimally sensitive for a diagnosis of probable PTSD (Prins et al., 2016). Post-traumatic stress was measured at T_1_ (6 months post-baseline).

### 3.4 Covariates

We selected confounders using a theory-driven approach, informed by a literature review and construction of a directed acyclic graph. We included the following sociodemographic and contextual variables as potential confounders: age (Collinson and Reed, 2018, Schwartz et al., 2022), gender (male, non-male) (Hepburn et al., 2020, Moore and Liang, 2020), race and ethnicity (non-Latinx Black, non-Latinx white, Latinx, other) (Hepburn et al., 2020, Parast et al., 2022), education (less than high school, high school, more than high school) (Desmond and Gershenson, 2017, Hong et al., 2007), and incarceration in the two years preceding the baseline survey (yes/no) (Geller and Curtis, 2011, Mallik-Kane et al., 2018). We conceptualize race and ethnicity as a proxy for exposure to racism in housing markets and healthcare settings (Quillian et al., 2020, Reskin, 2012, Yearby, 2018).

In progressive adjustment models, we controlled for additional health-related variables: health insurance status (insured/uninsured) (Zewde et al., 2019, Zhou et al., 2017), number of comorbidities (continuous, from a select-all-that-apply list of 20 common conditions) (Fisher et al., 2021, Cabral et al., 2019, Hajat et al., 2021), and whether a participant reported ever being diagnosed with a mental health condition (yes/no) (Desmond, 2016, LaCalle et al., 2013, Padgett, 2020). All theorized confounders were measured at T_0_ (baseline). We further elaborate on the rationale for including these covariates in Supplemental Table 2.

## 4 Analysis

### 4.1 Main analyses

We first described sample characteristics, using chi-square tests and t-tests to examine differences by exposure status. Next, we used separate logistic regression models to examine whether odds of ED use were higher among those who experienced an LRFM than those did not experience such a move. To assess short-term and medium-term associations between LRFM and ED use, we examined ED use in separate models at T_1_ and T_2_.

We used progressive adjustment to examine whether inclusion of theorized confounders, measured at baseline, altered the relationship between LRFM and ED use. To examine whether effects of legal eviction on ED use differed from effects of LRFM, more broadly, we ran separate models examining legal eviction as our exposure.

To assess whether our findings might be due to differential retention, we used logistic regression to examine whether reporting a legal eviction, landlord-related forced move, or ≥3 post-traumatic stress symptoms was associated with loss-to-follow-up at T_1_ or T_2_. We determined that none of these factors were significant predictors of loss-to-follow-up at T_1_ or T_2_.

### 4.2 Mediation analyses

We followed a four-step (Baron and Kenny, 1986, Baron and Kenny, 1986) approach to assess whether experiencing post-traumatic stress symptoms (T_1_) mediated the association between LRFM (T_0_) and ED use (T_2_). Specifically, we sought to determine if there was a correlation between: (1) LRFM and ED use; (2) LRFM and PTSD; and (3) PTSD and ED use, controlling for LRFM. Finally, we (4) sought to determine whether the correlation between LRFM and ED use decreased after adjusting for PTSD.

To obtain an adjusted estimate of the proportionate mediated effect of post-traumatic stress symptoms, we obtained a two-way decomposition of the total effect of LRFM on ED use, expressed as a sum of the natural direct effect and the natural indirect effect mediated through post-traumatic stress symptoms. We adjusted for sociodemographic (age, gender, education, race and ethnicity, and incarceration history) and other health-related (insurance status, past mental health diagnosis, and number of common comorbidities) confounders. To assess the statistical significance of indirect effects, we used bootstrapping of 5,000 samples (Preacher and Hayes, 2004).

Descriptive analyses and logistic regression were conducted using STATA 17.0/BE (StataCorp, 2021). Mediation analyses were conducted using the CausalMed procedure in SAS 9.4 (SAS Institute Inc., 2013*).*

## 5 Results

### 5.1 Descriptive statistics

Table 1 illustrates participants’ sociodemographic characteristics at baseline. Participants’ mean age was 45.7 (sd: 11.6). Nearly-two-thirds of participants identified as being Black (63.3 %); 18 % identified as non-Latinx white and 15.9 % identified as Latinx. Over three-fifths of participants were male (61.1 %). One-fifth of participants had less than a high school education (21.6 %). Nearly all (96.1 %) participants had some form of health insurance.

**Table 1:** Participant baseline characteristics, stratified by landlord-related forced move (LRFM) status at baseline.

Variable (timing of measurement)	No LRFM (n = 222)		LRFM (n = 61)		Test statistic	
Categorical	n	%	n	%	*X^2^*	*p*
Emergency dept. use at 0–6 months post-baseline (T_1_)						
*Yes*	40	18 %	21	34 %	*7.62*	<0.01
*No*	182	82 %	40	66 %		
Emergency dept. use at 6–12 months post-baseline (T_2_)						
*Yes*	39	18 %	26	43 %	*16.98*	<0.01
*No*	183	82 %	35	57 %		

PC-PTSD-5 Score at 6 months post-baseline (T_1_)						
≥*3 (suggestive of potential PTSD diagnosis)*	38	17 %	23	37 %	12.00	<0.01
*< 3*	184	83 %	38	63 %		
Gender (T_0_)						
*Male*	139	63 %	34	56 %	*0.95*	*0.33*
*Non-male*	83	37 %	27	44 %		

Race and ethnicity (T_0_)						
*Black (non-Latinx)*	139	63 %	40	66 %	*4.64*	*0.20*
*White (non-Latinx)*	38	17 %	13	21 %		
*Latinx*	40	18 %	5	8 %		
*Other*	5	2 %	3	5 %		
Education (T_0_)						
*Less than High School / GED*	50	22 %	11	18 %	*0.61*	*0.74*
*High School / GED*	104	47 %	31	51 %		
*More than High School / GED*	68	31 %	19	31 %		

Incarceration in past 2 years (T_0_)						
*Yes*	95	43 %	30	49 %	*0.79*	*0.37*
*No*	127	57 %	31	51 %		

Had health insurance (T_0_)						
*Yes*	213	96 %	59	97 %	*0.08*	*0.78*
*No*	9	4 %	2	3 %		

Past mental health diagnosis (T_0_)						
*Yes*	115	52 %	42	69 %	*5.63*	*0.02*
*No*	107	48 %	19	31 %		
				
Continuous	Mean (SD)		Mean (SD)		*T*	*p*
Age (years) (T_0_)	46.3 (11.1)		43.2 (12.8)		*1.83*	*0.07*
Number of common comorbidities (T_0_)	1.82 (1.94)		2.28 (2.58)		*−1.52*	*0.13*

At baseline, 61 participants (21.6 %) reported an LRFM in the past two years. Of these, 31 had experienced a legal eviction within the past two years. Participants who reported an LRFM were more likely to report a prior mental health diagnosis (69 % vs 52 %, p =.02) and were younger, on average, than those who did not report such moves (43.2 vs 46.3, p =.07).

At T_1_, 61 participants (21.6 %) reported that they had used a hospital emergency department in the last six months, and 61 participants (21.6 %) responded “yes” to three or more PC-PTSD-5 screening questions. At T_2_, 65 participants (23.0 %) reported ED use in the past six months. 33 participants reported ED use at both T_1_ and T_2_.

### 5.2 Multivariable logistic regression

Table 2 shows results of logistic regression models of ED use on LRFM status. In bivariate analyses, participants who reported an LRFM had 2.39 times the odds of ED use at T_1_ (95 % CI: 1.27–4.48), compared to those without an LRFM. After adjustment for potential confounders, those reporting an LRFM had 2.06 times the odds of ED use at T_1_ (95 % CI: 1.04–4.06). The association between LRFM and ED use was larger in unadjusted and adjusted models examining ED use at T_2_ (OR = 3.49, 95 % CI: 1.89–6.44; AOR = 3.05, 95 % CI: 1.59–5.88).

**Table 2:** Unadjusted and adjusted odds ratios (OR) and 95% confidence intervals (CI) from logistic regression models of lagged ED use on landlord-related forced move and potential confounders.

	ED use 0–6 months post-baseline (T_1_)				ED use 6–12 months post-baseline (T_2_)			
		95 % CI				95 % CI		
	OR	Low	High	P-value	OR	Low	High	P-value
Model 1	2.39	1.27	4.48	*0.01*	3.49	1.89	6.44	*0.00*
Model 2	2.44	1.26	4.70	*0.01*	3.56	1.88	6.73	*0.00*
Model 3	2.20	1.13	4.31	*0.02*	3.28	1.72	6.26	*0.00*
Model 4	2.06	1.04	4.06	*0.04*	3.05	1.59	5.88	*0.00*

*Model 1: unadjusted.

*Model 2: adjusted for age, gender, race/ethnicity, education, and recent incarceration history.

*Model 3: adjusted for Model 2, health insurance status and number of diagnosed common comorbidities.

*Model 4: adjusted for Model 3, and history of mental health diagnosis.

Table 3 shows the results of logistic regression models of ED use on legal eviction. In bivariate analyses, legal eviction was positively associated with ED use at T_1_ (OR = 2.22, 95 % CI: 1.00–4.94) and T_2_ (OR = 4.42, 95 % CI: 2.05–9.55). Adjusted models examining ED use at T_1_ were not significant (OR = 1.61, 95 % CI: 0.68–3.81). However, in fully adjusted models, legal eviction was associated with ED use at T_2_ (OR 3.58, 95 % CI: 1.58–8.10).

**Table 3:** Unadjusted and adjusted odds ratios (OR) and 95% confidence intervals (CI) from logistic regression models of lagged ED use on legal eviction and potential confounders.

	ED use 0–6 months post-baseline (T_1_)				ED use 6–12 months post-baseline (T_2_)			
		95 % CI				95 % CI		
	OR	Low	High	P-value	OR	Low	High	P-value
Model 1	2.22	1.00	4.94	0.05	4.42	2.05	9.55	0.00
Model 2	1.92	0.84	4.43	0.12	4.16	1.88	9.22	0.00
Model 3	1.71	0.73	4.01	0.22	3.77	1.68	8.46	0.00
Model 4	1.61	0.68	3.81	0.28	3.58	1.58	8.10	0.00

*Model 1: unadjusted.

*Model 2: adjusted for age, gender, race/ethnicity, education, and recent incarceration history.

*Model 3: adjusted for Model 2, health insurance status and number of diagnosed common comorbidities.

*Model 4: adjusted for Model 3, and history of mental health diagnosis.

### 5.3 Mediation analyses

**Fig. 1:**
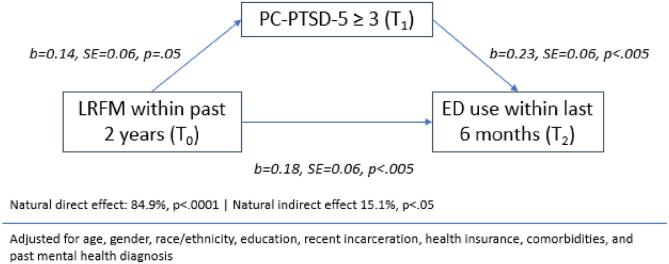
Mediation analysis demonstrating indirect and direct effects of landlord related forced moves on ED use, through post-traumatic stress symptoms.

Fig. 1 shows results of mediation analyses examining direct and indirect effects of LRFM on ED use. LRFMs were associated with experiencing symptoms of post-traumatic stress at T_1_ (*b* = 0.14, *SE* = 0.06, *p* <.05). Post-traumatic stress was positively associated with ED use at T_2_ (*b* = 0.23, *SE* = 0.06, *p* <.01). LRFM indirectly influenced ED use through post-traumatic stress (*b* = 0.03, *SE* = 0.02, *p* <.05). Post-traumatic stress accounted for 15.1 % of the total effect (*p* <.05).

**Fig. 2:**
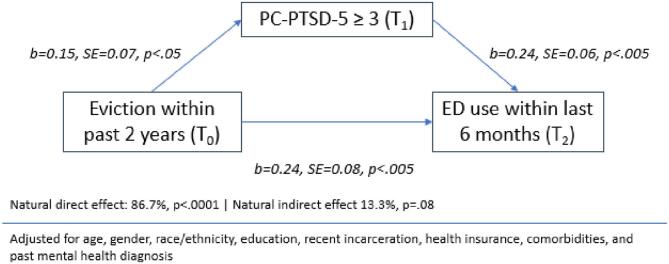
Mediation analysis demonstrating indirect and direct effects of legal eviction on ED use, through post-traumatic stress symptoms.

Fig. 2 shows results of mediation analyses examining direct and indirect effects of eviction on ED use. Findings were similar to Fig. 1, with the natural indirect effect of PTSD (T_1_) accounting for 13.3 % of the total effect. However, the indirect effect of PTSD was only marginally significant at a threshold of *p* <.05.

## 6 Discussion

While a growing body of literature demonstrates the adverse impacts of eviction on health, the relationships between eviction (and other landlord-related forced moves) and ED use are underexplored.

Accounting for sociodemographic and health-related confounders, we found that individuals who reported experiencing legal eviction or another landlord-related forced move within the past two years had higher odds of subsequent ED use than those who did not report such moves. We found that the associations between LRFMs (including, but not limited to, legal eviction) and ED use were similar to the associations between legal eviction and ED use. We found that the association between LRFM and ED use not only persisted but strengthened over time. And, in longitudinal mediation models, we found that experiencing symptoms of post-traumatic stress mediated the association between LRFMs and ED use.

These findings add to a small body of evidence that people with disruptions caused by forced moves are more likely to have poor mental health and use the ED than those without such disruptions (Collinson and Reed, 2018). Our results echo findings of two prior analyses that examined eviction and ED use (Collinson and Reed, 2018, Schwartz et al., 2022). Our research supports and extends these studies’ conclusions by examining forced moves inclusive of – but not limited to – legal eviction.

In addition, our findings regarding the mediating role of post-traumatic stress symptoms are aligned with prior studies examining impacts of eviction on stress (Hoke and Boen, 2021) and mental health (Desmond and Kimbro, 2015). Using a clinically-validated screening tool for PTSD (Prins et al., 2016), we extend prior studies’ findings by demonstrating that eviction and other LRFMs are associated with higher odds of experiencing post-traumatic stress symptoms. Our findings indicate that symptoms of post-traumatic stress are one possible factor associated with both forced moves and increased ED use. This relationship deserves further study. Understanding how trauma is associated with forced moves may help frame clinicians' approaches to caring for individuals experiencing such moves.

In recognition of the observed link between forced moves and ED use, we recommend that acute care settings incorporate standardized screening for risk and experience of forced moves into existing protocols. Prior studies have shown that EDs are often one of the first venues where people seek help after becoming homeless (O’Toole et al., 2007, Salhi et al., 2018). Despite this, in a recent national survey, just 60 % of hospitals and 28 % of outpatient practices self-reported that they routinely screen for housing insecurity (Fraze et al., 2019). Because forced moves may influence future healthcare use, efforts to improve screening for housing needs are necessary to facilitate referrals to supportive services – including social work, behavioral health, and/or medical-legal partnerships – that may promote housing stability, facilitate coping, and address ongoing health needs (Wallace, 2020, Holl et al., 2016, Taylor et al., 2016). Recognizing the high prevalence of post-traumatic stress symptoms among individuals using the ED who have also experienced an LRFM, we further recommend that organizational leaders partner with patients and providers to develop and implement trauma-informed approaches to screening and care delivery (Menschner and Maul, 2016). In doing so, organizational leaders should actively solicit providers’ insight regarding staffing and environmental factors that may impede effective implementation (Menschner and Maul, 2016).

More broadly, policy interventions are needed to address the rising – and inequitable – burden of eviction and forced moves, within and outside this study setting. In 2016, New Haven's legal eviction rate was over 4 %, approximately 75 % higher than the national eviction rate (2.3 %) (Eviction Lab, 2018a, Eviction Lab, 2018b). Like other U.S. cities, New Haven faces a growing housing affordability crisis; fair-market rent for a one-bedroom apartment is $1,181, nearly twice the rent ($624) considered affordable at a minimum wage of $13/hour (National Low Income Housing Coalition, 2021). To ease cost burdens and reduce eviction filings, investments are needed to increase the stock and accessibility of safe and affordable housing (by creating new housing units, renovating existing uninhabitable units, and ensuring ongoing affordability for low-income renters). Parallel investments are needed to make existing units more affordable (e.g., through housing voucher programs) and to prevent eviction among unstably housed renters (Desmond, 2020, National Low Income Housing Coalition, 2021). Estimates suggest that as many as 90 % of tenants undergoing legal eviction proceedings lack legal counsel, which places them at disproportionate risk of eviction (Greenberg et al., 2016, Holl et al., 2016, Sandefur, 2010). In June 2021, Connecticut became one of the first states to pass legislation establishing a right to free legal counsel for low-income tenants undergoing eviction proceedings (Soule, 2021). Further attention is needed to understand the health-related impacts of such programs and how they can be optimally implemented. Simultaneously, supportive services are needed to prevent forced moves occurring outside legal processes. In sum, multi-component policy interventions are urgently needed to improve housing affordability and prevent forced moves, especially as eviction moratoria are withdrawn (Benfer et al., 2020, Eviction Lab, 2022).

### 6.1 Strengths, limitations, and directions for future research

This study has several limitations. First, because we were unable to obtain data on participants’ ED use prior to eviction, we cannot infer a causal relationship between eviction and ED use (as higher ED use among those who experienced eviction may have been present before the study period). Relatedly, because we could not assess the degree to which post-traumatic stress symptoms preceded eviction, we cannot infer a causal relationship. Secondly, while we adjust for numerous relevant sociodemographic and other health-related confounders, we were unable to account for all factors that may influence one’s likelihood of eviction and ED use (e.g., substance use), which may influence the strength of the observed relationship. Thirdly, our study relies on a non-random sample of low-income individuals, approximately half of whom had recently been released from prison before the baseline survey. Consequently, our sample features a high proportion of Black men, formerly incarcerated individuals, and individuals with a history of mental health conditions. While this sampling design may limit generalizability, our sample is similar in many ways to individuals facing the highest risk of forced moves. Thus, our findings have direct relevance to clinicians, social service providers, and policymakers seeking to understand and address structural determinants of health inequities. In addition, while this study includes a more comprehensive measure of landlord-related forced moves than many prior studies, we were unable to examine whether repeated forced moves were associated with greater risk of PTSD or ED use. Lastly, while we examined ED use at multiple time points, use of a dichotomous outcome precludes examination of repeated ED use, which may be relevant to payers and providers.

Still, this study has several strengths. We provide insight into the association between ED use and informal evictions, which are more common than legal evictions but infrequently examined in empiric literature (Gromis and Desmond, 2021). Such insight is critical, particularly given recent reporting which suggests that informal evictions have persisted despite policy interventions (e.g., eviction moratoria) that have constrained landlords’ ability to remove tenants via legal processes (Groover, 2021, Salman, 2021). We used a longitudinal design and controlled for sociodemographic and other health-related factors that may confound the association between forced moves and ED use. Lastly, using a clinically validated screener for post-traumatic stress symptoms, we demonstrate forced moves’ association with a specific mental health condition that is relevant to clinical practice but overlooked in extant research.

Future studies may build on this work by examining other factors – including health, residential displacement, and preventative healthcare disruption – that may mediate the association between forced moves and ED use. Future research may also examine impacts of forced moves on ED use using longitudinal designs with representative samples. Policymakers and researchers can facilitate such research by including measures of eviction and other forced moves in national health-related surveys. Lastly, to better estimate downstream costs of forced moves, future analyses may also assess relationships between forced moves and continuous measures of ED use, including frequency and cost.

## 7 Conclusions

This study fills gaps in research on housing instability and health by examining how legal eviction and other forced moves are associated with ED use over time. We found that eviction and other landlord-related forced moves were associated with higher odds of post-traumatic stress and subsequent ED use. These results add to a growing body of research on how eviction and forced moves influence health. In response to these findings, we recommend that providers and healthcare organizations work to implement trauma-informed approaches to care, alongside routine screening for housing-related needs. In addition, considering projected increases in eviction and other forced moves, we recommend that policymakers further intervene to prevent evictions and increase access to safe and affordable housing.

## CRediT authorship contribution statement

**Patrick D. Smith:** Conceptualization, Methodology, Writing – original draft, Writing – review & editing. **Allison K. Groves:** Conceptualization, Methodology, Writing – review & editing. **Brent A. Langellier:** Conceptualization, Methodology, Writing – review & editing. **Danya E. Keene:** Conceptualization, Writing – review & editing. **Alana Rosenberg:** Conceptualization, Writing – review & editing, Investigation, Project administration. **Kim M. Blankenship:** Conceptualization, Writing – review & editing, Supervision, Funding acquisition.

## Declaration of Competing Interest

The authors declare that they have no known competing financial interests or personal relationships that could have appeared to influence the work reported in this paper.
