# Supplementary material for: Eviction, post-traumatic stress, and emergency department use among low-income individuals in New Haven, CT
Source: Prev Med Rep. 2022 Aug 17;29:101956. doi: 10.1016/j.pmedr.2022.101956 (PMC9502672; doi:10.1016/j.pmedr.2022.101956)
Supplement: Supplementary data 1 [file mmc1.docx]

| **Supplemental Table 1: Characteristics of Included vs. Excluded Participants** | | | | | | | | |
| --- | --- | --- | --- | --- | --- | --- | --- | --- |
| **Variable** | **JustHouHS Sample (n=400)** | | **Included**  **(n=283)** | | **Excluded**  **(n=117)** | | **Test Statistic** | |
|  | *N* | *Col. %* | *N* | *Col. %* | *N* | *Col. %* | *X^2^* | *p* |
| **Legal eviction in last 2 years** |  |  |  |  |  |  |  |  |
| *No* | 362 | 91% | 252 | 89% | 110 | 94% | 2.34 | 0.123 |
| *Yes* | 38 | 10% | 31 | 11% | 7 | 6% |  |  |
| **LRFM in last 2 years** |  |  |  |  |  |  |  |  |
| *No* | 314 | 79% | 222 | 78% | 92 | 79% | 0.00 | 0.967 |
| *Yes* | 86 | 22% | 61 | 22% | 25 | 21% |  |  |
| **ED Use 0-6 months post-baseline** |  |  |  |  |  |  |  |  |
| *Missing* |  |  |  |  | 84 | |  |  |
| *No* | 327 | 82% | 222 | 78% | 21 | 64% | 3.65 | 0.06 |
| *Yes* | 73 | 18% | 61 | 22% | 12 | 36% |  |  |
| **ED Use 6-12 months post-baseline** |  |  |  |  |  |  |  |  |
| *Missing* |  |  |  |  | 91 | |  |  |
| *No* | 326 | 82% | 218 | 77% | 17 | 65% | 1.77 | 0.18 |
| *Yes* | 74 | 19% | 65 | 23% | 9 | 35% |  |  |
| **PC-PTSD-5 score** |  |  |  |  |  |  |  |  |
| *Missing* |  |  |  |  | 82 | |  |  |
| *< 3* | 333 | 83% | 222 | 78% | 29 | 83% | 0.36 | 0.55 |
| $\geq$*3 (suggestive of potential PTSD)* | 67 | 17% | 61 | 22% | 6 | 17% |  |  |
| **Gender** |  |  |  |  |  |  |  |  |
| *Non-male* | 129 | 32% | 110 | 39% | 19 | 16% | 19.40 | 0.00 |
| *Male* | 271 | 68% | 173 | 61% | 98 | 84% |  |  |
| **Race and Ethnicity** |  |  |  |  |  |  |  |  |
| *Non-Latinx white* | 88 | 22% | 51 | 18% | 37 | 32% | 9.69 | 0.02 |
| *Non-Latinx black* | 237 | 59% | 179 | 63% | 58 | 50% |  |  |
| *Latinx* | 63 | 16% | 45 | 16% | 18 | 15% |  |  |
| *Other* | 12 | 3% | 8 | 3% | 4 | 3% |  |  |
| **Education** |  |  |  |  |  |  |  |  |
| *Less than HS/GED* | 94 | 24% | 61 | 22% | 33 | 28% | 2.89 | 0.24 |
| *HS/GED* | 191 | 48% | 135 | 48% | 56 | 48% |  |  |
| *More than HS/GED* | 115 | 29% | 87 | 31% | 28 | 24% |  |  |
| **Incarceration in past 2 years** |  |  |  |  |  |  |  |  |
| *No* | 190 | 48% | 158 | 56% | 32 | 27% | 26.92 | 0.00 |
| *Yes* | 210 | 53% | 125 | 44% | 85 | 73% |  |  |
| **Had health insurance** |  |  |  |  |  |  |  |  |
| *No* | 16 | 4% | 11 | 4% | 5 | 4% | 0.03 | 0.86 |
| *Yes* | 384 | 96% | 272 | 96% | 112 | 96% |  |  |
| **Mental health diagnosis** |  |  |  |  |  |  |  |  |
| *No* | 179 | 45% | 126 | 45% | 53 | 45% | 0.02 | 0.89 |
| *Yes* | 221 | 55% | 157 | 55% | 64 | 55% |  |  |
|  | *Mean* | *SD* | *Mean* | *SD* | *Mean* | *SD* | *t* | *p* |
| **Age** | 44.8 | 11.6 | 45.6 | 11.6 | 42.9 | 11.7 | -2.15 | 0.03 |
| **Number of common comorbidities** | 1.8 | 2.0 | 1.9 | 2.1 | 1.5 | 1.8 | -1.84 | 0.07 |

| **Supplemental Table 2: Inclusion of Covariates** | |
| --- | --- |
| **Potential Confounder** | **Rationale** |
| Gender | Past research has established that men are less likely than women to experience eviction.^1^ In addition, recent data from the Agency for Healthcare Research and Quality (AHRQ) indicates that men account for lower share of emergency department visits than women.^2^ |
| Race and Ethnicity | Past research has established that Black and Latinx individuals face disproportionately high rates of eviction filings.^1^ In addition, past research suggests that emergency department use is more common among Black and Latinx individuals than among non-Latinx white individuals.^3^ A wider body of literature indicates that such differences may emerge as a product of repeated exposure to racism in housing markets and healthcare settings.^4–6^ |
| Age | Past research examining the relationship between eviction and acute care use has accounted for the influence of age.^7,8^ |
| Education | Education is commonly understood to influence socioeconomic status, and has been included in past studies examining correlates of both eviction^9^ and ED use.^10^ |
| Incarceration | Individuals who have recently been incarcerated are more likely to experience subsequent housing insecurity,^11^ including high degrees of residential turnover (which may occur as a result of eviction filings). Incarceration disrupts access to outpatient healthcare services and may increase the likelihood that individuals use the emergency department as a usual source of care after release.^12^ |
| Health insurance status | Health insurance is a means of financial protection that may protect individuals from accruing unmanageable medical bills (which could contribute to non-payment of rent and, subsequently, eviction). Recent research indicates that Medicaid expansion under the Affordable Care Act has been linked to declines in eviction rates.^13^ In addition, while some data indicate that health insurance is not directly linked to increased emergency department use, having insurance does influence access to and use of preventative healthcare services, which may affect one’s overall health status, including the management of chronic conditions.^14^ |
| Comorbidities | Multimorbidity is associated with increased acute care use,^15^ and it is likely that having multiple health conditions could increase one’s socioeconomic risk of experiencing eviction (due to higher medical bills, increased likelihood of experiencing an unanticipated and costly health emergency, and/or increased difficulty maintaining employment.^16,17^ |
| Mental health diagnosis | Past research has established a bidirectional relationship between mental health and housing instability,^18^ and prior ethnographic work suggests that the presence of a mental health condition may increase one’s risk of being evicted.^19^ The presence of a mental health condition is also a known driver of acute care utilization.^20^ |
| 1. Hepburn P, Louis R, Desmond M. Racial and Gender Disparities among Evicted Americans. *Sociol Sci*. 2020;7:649-662. doi:10.15195/v7.a27  2. Moore B, Liang L. *Statistical Brief: Costs of Emergency Department Visits in the United States, 2017*. Agency for Healthcare Research and Quality; 2020:14. https://hcup-us.ahrq.gov/reports/statbriefs/sb268-ED-Costs-2017.pdf  3. Parast L, Mathews M, Martino S, Lehrman WG, Stark D, Elliott MN. Racial/Ethnic Differences in Emergency Department Utilization and Experience. *J Gen Intern Med*. 2022;37(1):49-56. doi:10.1007/s11606-021-06738-0  4. Reskin B. The Race Discrimination System. *Annu Rev Sociol*. 2012;38(1):17-35. doi:10.1146/annurev-soc-071811-145508  5. Yearby R. Racial Disparities in Health Status and Access to Healthcare: The Continuation of Inequality in the United States Due to Structural Racism. *Am J Econ Sociol*. 2018;77(3-4):1113-1152. doi:10.1111/ajes.12230  6. Quillian L, Lee JJ, Honoré B. Racial Discrimination in the U.S. Housing and Mortgage Lending Markets: A Quantitative Review of Trends, 1976–2016. *Race Soc Probl*. 2020;12(1):13-28. doi:10.1007/s12552-019-09276-x  7. Collinson R, Reed D. The Effects of Evictions on Low-Income Households. :82.  8. Schwartz GL, Feldman JM, Wang SS, Glied SA. Eviction, Healthcare Utilization, and Disenrollment Among New York City Medicaid Patients. *Am J Prev Med*. 2022;62(2):157-164. doi:10.1016/j.amepre.2021.07.018  9. Desmond M, Gershenson C. Who gets evicted? Assessing individual, neighborhood, and network factors. *Soc Sci Res*. 2017;62:362-377. doi:10.1016/j.ssresearch.2016.08.017  10. Hong R, Baumann BM, Boudreaux ED. The emergency department for routine healthcare: Race/ethnicity, socioeconomic status, and perceptual factors. *J Emerg Med*. 2007;32(2):149-158. doi:10.1016/j.jemermed.2006.05.042  11. Geller A, Curtis MA. A Sort of Homecoming: Incarceration and the housing security of urban men. *Soc Sci Res*. 2011;40(4):1196-1213. doi:10.1016/j.ssresearch.2011.03.008  12. Health Care After Incarceration. Urban Institute. Accessed May 30, 2022. https://www.urban.org/research/publication/health-care-after-incarceration  13. Zewde N, Eliason E, Allen H, Gross T. The Effects of the ACA Medicaid Expansion on Nationwide Home Evictions and Eviction-Court Initiations: United States, 2000-2016. *Am J Public Health*. 2019;109(10):1379-1383. doi:10.2105/AJPH.2019.305230  14. Zhou RA, Baicker K, Taubman S, Finkelstein AN. The Uninsured Do Not Use The Emergency Department More—They Use Other Care Less. *Health Aff (Millwood)*. 2017;36(12):2115-2122. doi:10.1377/hlthaff.2017.0218  15. Fisher KA, Griffith LE, Gruneir A, et al. Effect of socio-demographic and health factors on the association between multimorbidity and acute care service use: population-based survey linked to health administrative data. *BMC Health Serv Res*. 2021;21(1):62. doi:10.1186/s12913-020-06032-5  16. Hajat C, Siegal Y, Adler-Waxman A. Clustering and Healthcare Costs With Multiple Chronic Conditions in a US Study. *Front Public Health*. 2021;8. Accessed June 2, 2022. https://www.frontiersin.org/article/10.3389/fpubh.2020.607528  17. Cabral GG, Dantas de Souza AC, Barbosa IR, Jerez-Roig J, Souza DLB. Multimorbidity and Its Impact on Workers: A Review of Longitudinal Studies. *Saf Health Work*. 2019;10(4):393-399. doi:10.1016/j.shaw.2019.08.004  18. Padgett DK. Homelessness, housing instability and mental health: making the connections. *BJPsych Bull*. 2020;44(5):197-201. doi:10.1192/bjb.2020.49  19. Desmond M. *Evicted: Poverty and Profit in the American City*. Broadway books; 2016.  20. LaCalle EJ, Rabin EJ, Genes NG. High-Frequency Users of Emergency Department Care. *J Emerg Med*. 2013;44(6):1167-1173. doi:10.1016/j.jemermed.2012.11.042 | |
